# A case of placental trisomy 18 mosaicism causing a false negative NIPT result

**DOI:** 10.1186/s13039-017-0341-5

**Published:** 2017-10-27

**Authors:** Jiexia Yang, Yiming Qi, Fangfang Guo, Yaping Hou, Haishan Peng, Dongmei Wang, Haoxin OY, Aihua Yin

**Affiliations:** 1 Prenatal Diagnosis Centre, Guangdong Women and Children Hospital, Guangzhou, Guangdong 511400 China; 2 Maternal and Children Metabolic-Genetic Key Laboratory, Guangdong Women and Children Hospital, Guangzhou, Guangdong 511400 China

**Keywords:** The non-invasive prenatal testing (NIPT), Cell-free DNA (cfDNA), False negative, Placental mosaicism

## Abstract

**Background:**

The non-invasive prenatal testing that evaluates circulating cell free DNA, and has been established as an additional pregnancy test for detecting the common fetal trisomies 21, 18 and 13 is rapidly revolutionizing prenatal screening as a result of its increased sensitivity and specificity. However, false positive and false negative results still exist.

**Case presentation:**

We presented a case in which the non-invasive prenatal testing results were normal at 15 gestational age (GA), but an ultrasound examination at 30GA showed that the fetus had heart abnormalities, and the third trimester ultrasound at 33GA noted multiple anomalies including a 3.0 mm ventricular septal defect. Along with cordocentesis at 33GA, the cord blood sample cytogenetics analysis showed a mos 47,XN,+18[61]/46,XN[39] T18 karyotype. Six placental biopsies confirmed that the chromosome 18 placenta chimerism ratio had changed from 33% to 72%. Ultimately, the pregnancy was interrupted at 34GA.

**Conclusions:**

We presented this case to highlight the need to clearly explain false positive or false negative results to patients. We believe that this information will also influence the development of future diagnostic test methodologies.

## Background

Non-invasive prenatal testing (NIPT) which was established as an additional pregnancy test for detecting of the common fetal trisomies 21 (T21), 18 (T18) and 13 (T13), is rapidly becoming a common clinical practice [1]. It evaluates circulating cell-free DNA (cfDNA) as early as 9 gestational age (GA). These DNA fragments are derived from apoptotic placental cytotrophoblast cells [2]. Cell-free fetal DNA (cffDNA) can be described quantitatively as the fetal DNA fraction, and is determined by the ratio of the absolute concentration of cffDNA to the absolute concentration of total (maternal and fetal) cfDNA [3]. NIPT has used for several years as part of prenatal care to screen high-risk patients for fetal aneuploidy, and it has been used increasingly in clinical practice. The pooled sensitivities in the selected high-risk pregnant population were 0.998 (95% CI 0.981 to 0.999), 0.977 (95% CI 0.958 to 0.987) for T21 and T18, respectively. Pooled sensitivity for T13 sensitivity was closer to 0.900. The pooled specificity in the high-risk population for trisomies 21, 18, and 13 is 0.999 (95% 0.998 to 0.999) [4, 5]. However, false positive and false negative results till exist, as cffDNA comes from apoptotic placental trophoblast cells [6]. Therefore, the results may not always represent the actual fetal karyotype in all cases. In the vast majority of pregnancies, although the genetic component between the placental and fetal tissue is identical, false positive or false negative results still exist due to confined placental mosaicism (CPM) [7, 8]. Some positive NIPT results were finally confirmed to be false positive, and common reasons include placental mosaicism, vanishing twin or cotwin demise, fetal chromosome rearrangement, and maternal chromosome abnormalities or malignancy [9, 10]. In contrast, there is a small chance of a false negative result. The fact that cffDNA in the maternal plasma fraction originates from the cytotrophoblast explains a part of the discrepancies between NIPT results and the actual fetal karyotype. A low level of cffDNA fraction in maternal plasma can also result a false negative NIPT result [11].

Herein, we presented one case of a patient whose fetus tested “negative” for T18 by NIPT but was diagnosed as mos 47,XN,+18[61]/46,XN[39]. Our report suggests that some pregnant women display regional placental mosaicism, which is sufficient to cause a discrepancy between the NIPT and karyotyping results.

## Methods

The NIPT test was performed at 15 and 34GA by sequencing cfDNA from the maternal peripheral blood. Blood collection, cfDNA extraction, library construction and sequencing were performed according to the instructions of JingXin Fetal Chromosome Aneuploidy (T21, T18, T13) Testing Kits (CFDA registration permit No. 0153400300) [12].

Based on our previous study, we developed a technique that uses the read length to estimate the concentration of fetal cfDNA in maternal plasma by sequencing [12]. The fetal DNA concentration was calculated as a quality control, as described in Yin’s paper [12]. Combined GC-correction and Z-score testing methods were used to identify fetal autosomal aneuploidy for trisomy as described in Liao’s paper [13]. Z score range from −3 to 3 was considered to indicate a low risk for a trisomy chromosome [14]. A cord blood sample was taken at 33GA. A cord blood sample and six placental biopsies (three from the maternal side and three from the fetal side) were taken for analysis. DNA sequencing, cfDNA extraction, library construction and sequencing were performed through bioconductor sequencing platform [12]. The speculated chimeric proportion of T18 was calculated from the ratio of the samples to the control. For example, for sample 1, the speculated chimeric proportion = 2*(1–3.327%/2.856%) =33%.

## Case presentation

A 32-year-old healthy pregnant woman was referred to the Medical Genetic Centre of Guangdong Women and Children Hospital. Maternal serum screening at 12GA combined nuchal translucency measurement (2.4 mm) showed a high risk of fetal T21 at 1 in 190. The NIPT at 15GA showed that the fetal DNA fraction in the maternal plasma sample was 7.8% (normal NIPT result) and the Chromosome18 Z scores were 1.889 (Table 1). NIPT provided at 15GA gave a low risk result.

**Table 1:** NIPT results for the Case

Gestational Weeks	Unique reads/M	Fetal DNA Fraction	NIPT Z-scores			NIPT result
			Chromosome21	Chromosome18	Chromosome13	
15	3.25	7.80%	−0.466	1.889	1.369	Low Risk
34	3.36	16.6%	−1.491	5.500	−0.016	High Risk of T18

Z scores were calculated as previously described [12] with a normal range > −3 and <3

**Fig. 1:**
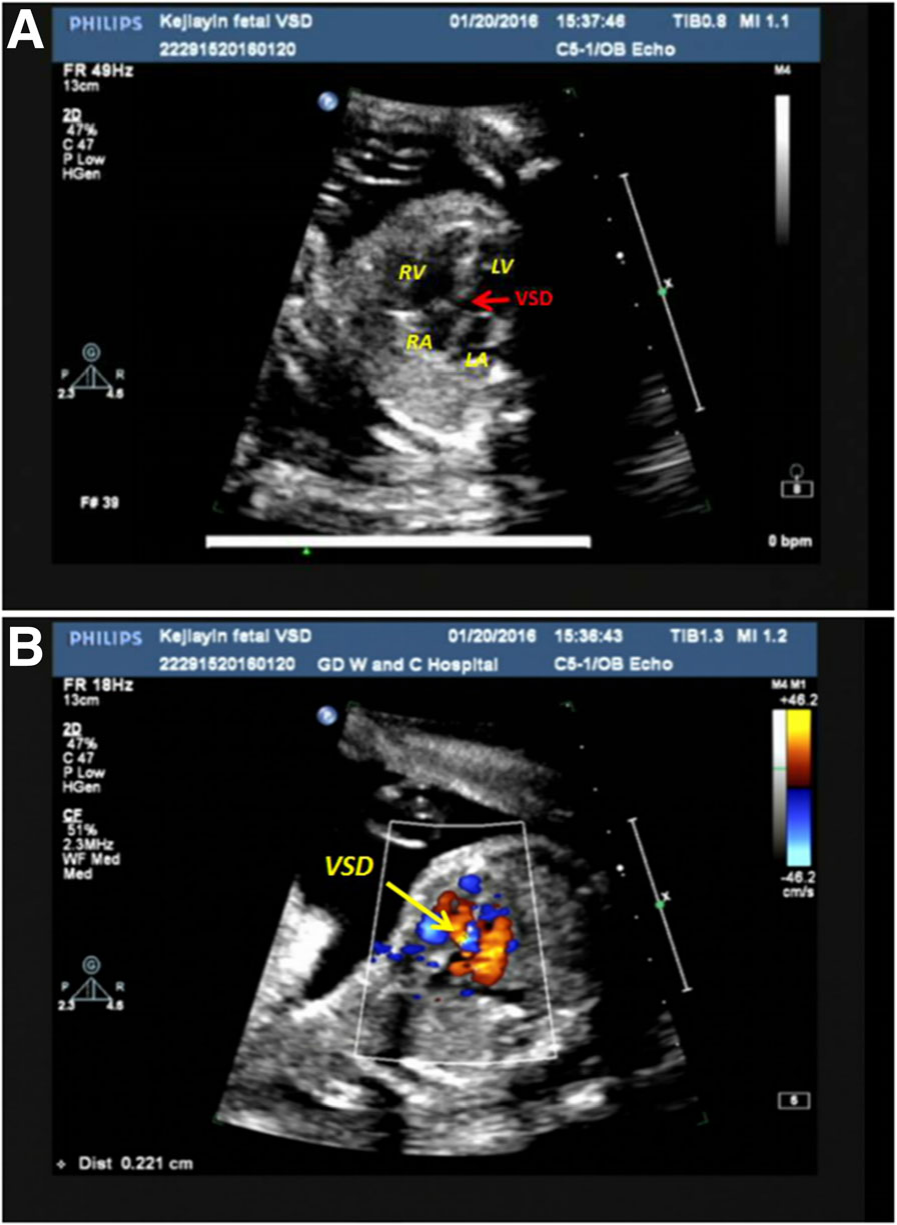
Ultrasound examination images. **a** Ultrasound examination result at 30wk. A ventricular septal defect for 2.2 mm was shown as the arrow in the image. Abbreviations: LA, left atrium; LV, left ventricle; RA, right atrium; RV, right ventricle; VSD, ventricular septal defect. **b** Ultrasound examination result at 33wk. A ventricular septal defect (VSD) for 3.0 mm was shown as the arrow in the image

As routine practice, ultrasound was conducted to monitor the developmental status of the fetus. The ultrasound examination at 24GA showed that the fetus displayed a defect in the ventricular compartment, which was confirmed by the ultrasound examination at 30 GA (Figure 1A). The ultrasound examination showed a 2.2 mm ventricular septal defect. At 33GA (Figure 1B), the patient was referred for further evaluation after a third trimester ultrasound revealed a 3.0 mm ventricular septal defect, and all four limbs were smaller than is observed at normal gestational weeks. For further counseling, the patient consented to have a cord blood sample taken by cordocentesis at 33GA. Cytogenetics analyses reported a karyotyping of mos 47,XN,+18[61]/46,XN[39] indicated that 61% of cells had trisomy 18 even though both parental karyotypes were normal. After genetic counseling and communicating with families, the pregnant woman opted to terminate her pregnancy at 34GA.

Maternal peripheral blood was collected for a confirmatory NIPT test before the pregnancy was interrupted at 34GA. Placental tissues were also retained. Six placental biopsies (three from the maternal side and three from the fetal side) were taken for sequencing. Placental biopsies confirmed that it was placental chimerism of chromosome18, with a chimeric ratio from 33% to 72% (Table 2). The chimeric ratios of the placental biopsies were consistently around 61%, which was the T18 mosaicism fetal umbilical cord blood type result. The NIPT reported Z-scores were −1.491, 5.500 and −0.016 for chromosome21, 18 and 13 respectily (Table 1), and the fetal fraction was 16.6%. These results confirmed that the fetus displayed T18 mosaicism, which indicated a false negative result for NIPT at 15GA.

**Table 2:** Sequencing results for six placental biopsies

Sample		Chromosome 18 Z scores	Chromosome 18 ratio	Speculated chimeric proportion of T18
maternal side	1	40.279	3.327%	33%
	2	40.066	3.322%	33%
	3	49.474	3.427%	40%
fetal side	4	48.311	3.420%	39%
	5	89.007	3.884%	72%
	6	44.263	3.370%	36%
Control		−2.58944	2.856%	/

Six placental biopsies (three from the maternal side, 1–3; three from the fetal side, 4–6) were taken for routine cytogenetics at 35GA

## Discussion and conclusions

Increasing amounts of evidence have shown that the circulating fetal DNA in maternal blood during pregnancy mainly originates from placental trophoblastic cells, although there are still small contributions from fetal tissues [2]. Since cfDNA was identified, it has been widely promoted for the development of NIPT [15]. However, many factors can still affect NIPT results; for example, the maternal weight can significantly decrease the fetal DNA fraction [16], which usually leads to a false negative result. Otherwise, 0.8–1% of cases are confirmed as placental mosaicism according to a large-scale evaluation of chorionic villi sampling (CVS) [17]. Placental mosaicism means that the cffDNA from cytotrophoblast cells has a different karyotype than that of the true fetal DNA [18]. Thus, in this study, we provided a false negative NIPT case caused by both fetal and placental mosaicism. Clinicians should be aware of this situation and patients should be informed of the possibility of discordant NIPT results.

In this case, of placental mosaicism, the fetal DNA fraction in the maternal plasma sample at 15GA was 7.8% (normal NIPT result) with a chromosome18 Z-score of 1.889. Examination of the placental tissue at six sites showed that T18 mosaicism, as measured by the ratio of trisomy to disomy 18, averaged approximately 42%. Further, the chimeric ratio varied from 32% to 72% at different sites, which suggested that there were significant regional variations in the T18 mosaicism in this placenta. However, this was consistent with the fetal umbilical cord blood karyotype results, which showed a T18 mosaicism ratio of 61%. Furthermore, at 15GA, the fetal DNA percentage was 7.8% due to the mosaicism, and the effective/perceived fetal fraction observed by the algorithm on the trisomy chromosome was (100%–42%)*7.8% = 4.52%. However, a trisomy with an actual fetal fraction of 4.52% is mathematically insufficient to detect the 42% placental T18 when using our NIPT test. The fetal DNA concentration increases with gestational weeks [19]. At 35GA, the increased fetal DNA fraction was sufficient to detect the mosaicism. Similar patterns of mosaicism were also reported in cases with false negative cfDNA screening results [8, 9, 20].

This report describes a case of a false negative cell-free DNA result for trisomy 18 due to fetal and placental mosaicism. To date, few NIPT reports have demonstrated that placental mosaicism [14, 21, 22] manifests quite differently across individual pregnancies in pregnant women. The existence of false negatives due to mosaicism is not unique, and information pertaining to these cases is still limited. Multicenter studies have determined the frequency of mosaicism to be approximately 1% [23].In most situations, the mosaic cell line is only found in the placenta and will lead to a normal fetal outcome [24]. However the frequency of false negative fetal test results with maternal serum cffDNA testing due to placental mosaicism or other fetal-placental discrepancies is still unknown [7]. How should unexpected false negative NIPT results be handled in clinical practice? On the one hand, it is necessary that patients receive pretest counseling and informed consent prior to NIPT screening. Patients should be aware of the potential for false positive and false negative results as well as discordant results due to differences between the fetally and parentally derived analyzed samples [7]. We hope to give clinicians a reference with this case. Clinicians should be cognizant of false negative results when the fetal DNA concentrations are relatively low. In contrast, as the frequency and level of various types of mosaicism are still limited, new and accurate documents are valuable to the diagnostic and medical community. It is necessary for the NIPT field to improve its sensitivity and specificity and reduce the incidence of discordant results. This information can not only contribute to the development of future screening test methodologies and algorithms, but can also help optimize the counseling and medical decision applied by medical practitioners.

## Abbreviations

cfDNA: Cell free DNA
cffDNA: Cell-free fetal DNA
CPM: Confined placental mosaicism
CVS: Chorionic villi sampling
GA: Gestational age
NIPT: Non-invasive prenatal testing
T18: Trisomy 18
